# Supplementary material for: The induction of peripheral trained immunity in the pancreas incites anti-tumor activity to control pancreatic cancer progression
Source: Nat Commun. 2022 Feb 9;13:759. doi: 10.1038/s41467-022-28407-4 (PMC8828725; doi:10.1038/s41467-022-28407-4)
Supplement: Supplementary file 1 — Supplementary Information [file 41467_2022_28407_MOESM1_ESM.pdf]

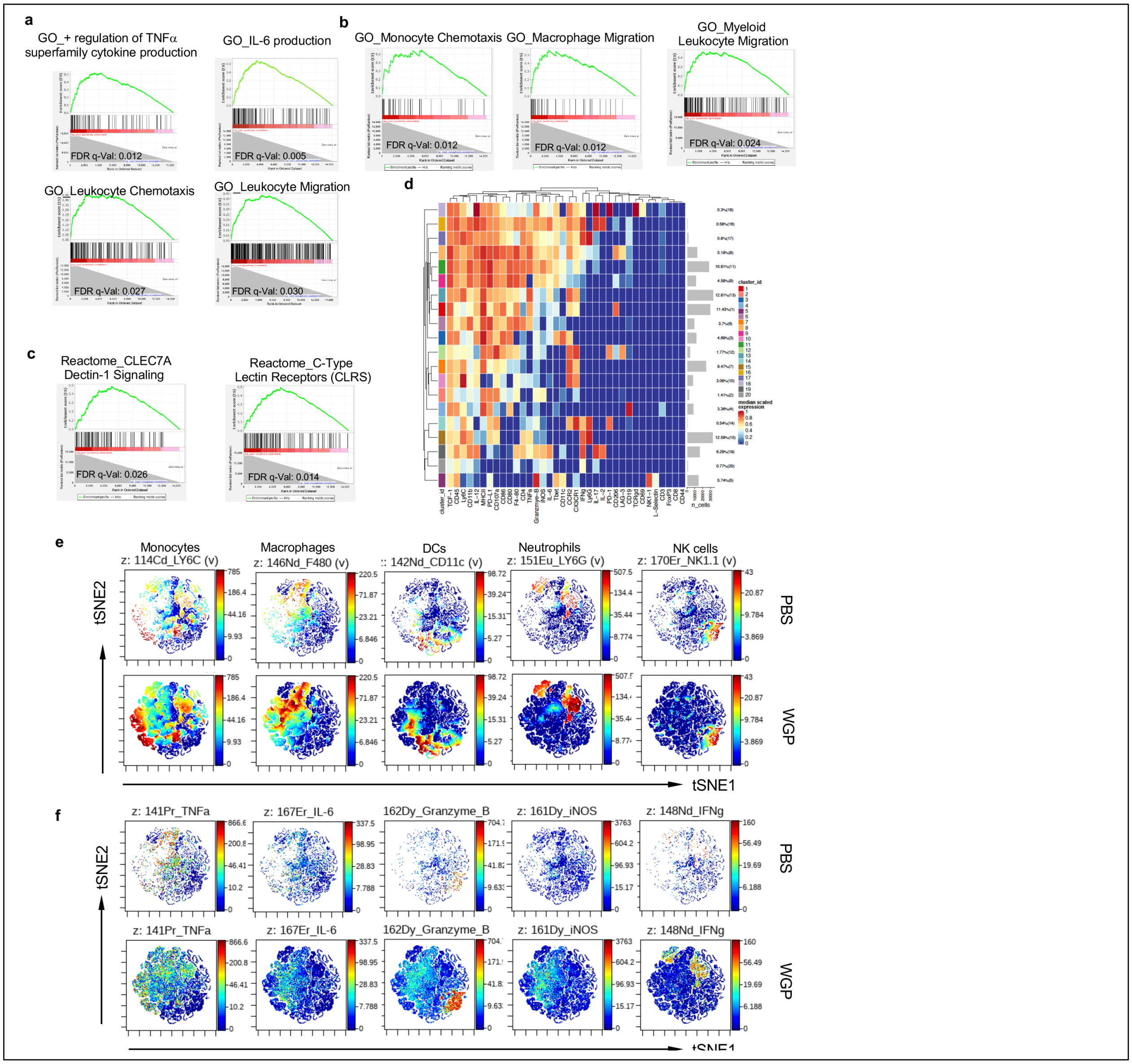

Supplementary Fig. 2: Characterization of the increased pancreatic CD11b$^+$ population using RNA-Seq and CyTOF

(a) Enrichment plots generated by Gene Set Enrichment Analysis (GSEA) for genes related to TNFα and IL-6 production, along with leukocyte chemotaxis and leukocyte migration in CD11b$^+$ cells from 7-day WGP-trained as compared to PBS mice. PBS was used as the control and compared to WGP. **(b)** GSEA for genes related to monocyte chemotaxis, macrophage migration, myeloid leukocyte migration in CD11b$^+$ cells from 7-day WGP trained as compared to PBS mice. **(c)** GSEA for genes related to CLEC7A Dectin-1 Signaling and C-type Lectin Receptor Signaling in CD11b$^+$ cells from 7-day WGP trained as compared to PBS mice. **(d)** Heatmap of the median scaled expression of the CyTOF markers in each cluster determined from cytof workflow. **(e)** viSNE plots of CD11b$^+$ pancreatic cells in PBS and 7-day WGP trained mice, made from CyTOF data, highlighting the specific expression of Ly6C, F4/80, CD11c, Ly6G and NK1.1 in order to indicate the relative location of the major immune-cell subsets and changes between the PBS and WGP treatment group. **(f)** viSNE plots of CD11b$^+$ pancreatic cells in PBS and 7-day WGP trained mice, made from CyTOF data, showing the expression of TNFα, IL-6, Granzyme-B, iNOs and IFNγ.

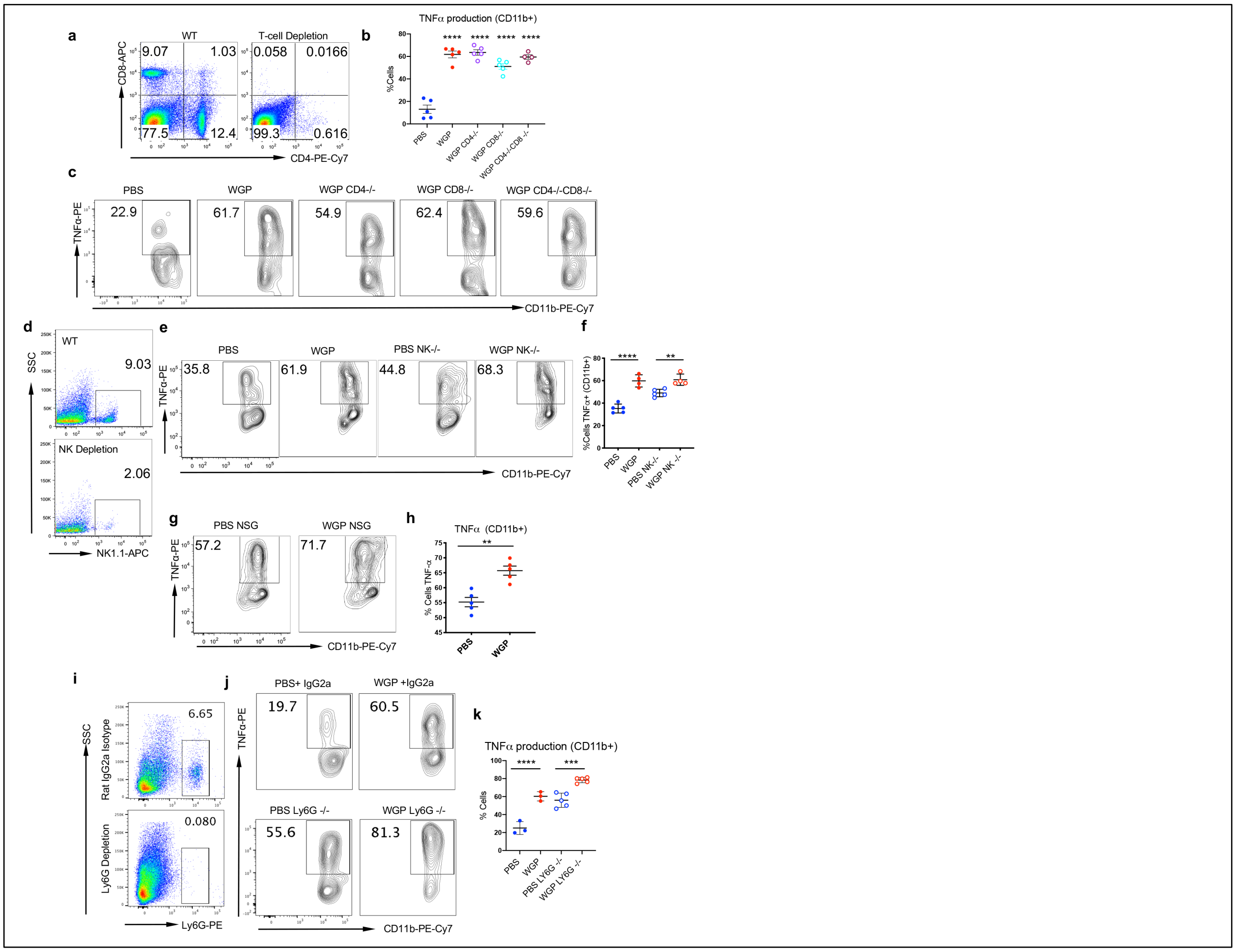

Supplementary Fig. 3: Adaptive immune cells, NK cells and neutrophils are not involved in the training of myeloid cells

(a) Representation of the T-cell depletion efficiency in the spleen of anti-CD-4 and cnti-CD-8 mAbs used *in vivo.* **(b, c)** Mice depleted of $CD4^+$ T-cells (n=5), $CD8^+$ T-cells (n=5) or both $CD4^+$ and $CD8^+$ T-cells (n=4) and WT mice (n=5) were given I.P. WGP and 7 days later the pancreatic cells from these mice and WT mice treated with PBS were restimulated with LPS. TNFα production in the $CD11b^+$ cells was measured by flow cytometry. Representative flow plots (**c**) and summarized data are shown (**b**). ****p<0.0001. **(d)** Mice were treated with an anti NK1.1 mAb to deplete NK cells and the depletion efficiency of NK cells in the pancreas was assessed. **(e, f)** NK-cell depleted mice and WT mice were treated with PBS or WGP and 7 days later the pancreatic cells from these mice were restimulated with LPS. TNFα production in the $CD11b^+$ cells was measured by flow cytometry. Representative flow plots (**e**) and summarized data are shown (**f**). PBS n=5, WGP n=4, PBS $NK^{-/-}$ n=5, WGP $NK^{-/-}$ n=4. **p=0.0064, ****p<0.0001. **(g, h)** NSG mice (n=5) were injected with PBS or WGP and 7 days later pancreatic cells were restimulated with LPS. TNFα production in the $CD11b^+$ cells was measured by flow cytometry. Representative flow plots (**g**) and summarized data are shown (**h**). **p=0.0014. **(i-k)** Mice treated with the anti-Ly6G mAb and isotype control were given either PBS or WGP and 7 days later pancreatic cells were restimulated with LPS. TNFα production in the $CD11b^+$ cells was measured by flow cytometry (PBS n=3, WGP n=3, PBS $NK^{-/-}$ n=5, WGP $NK^{-/-}$ n=5). Representative flow plots to show neutrophil depletion efficiency (**i**) and intracellular TNFα production (**j**) and summarized TNFα data are shown (**k**). ***p=0.0004, ****p<0.0001. Data are represented as mean ± SEM. A one-way ANOVA with multiple comparisons was used for **b**, **f** and **k** while an unpaired student's t-test was used for **h**. ns= not significant.

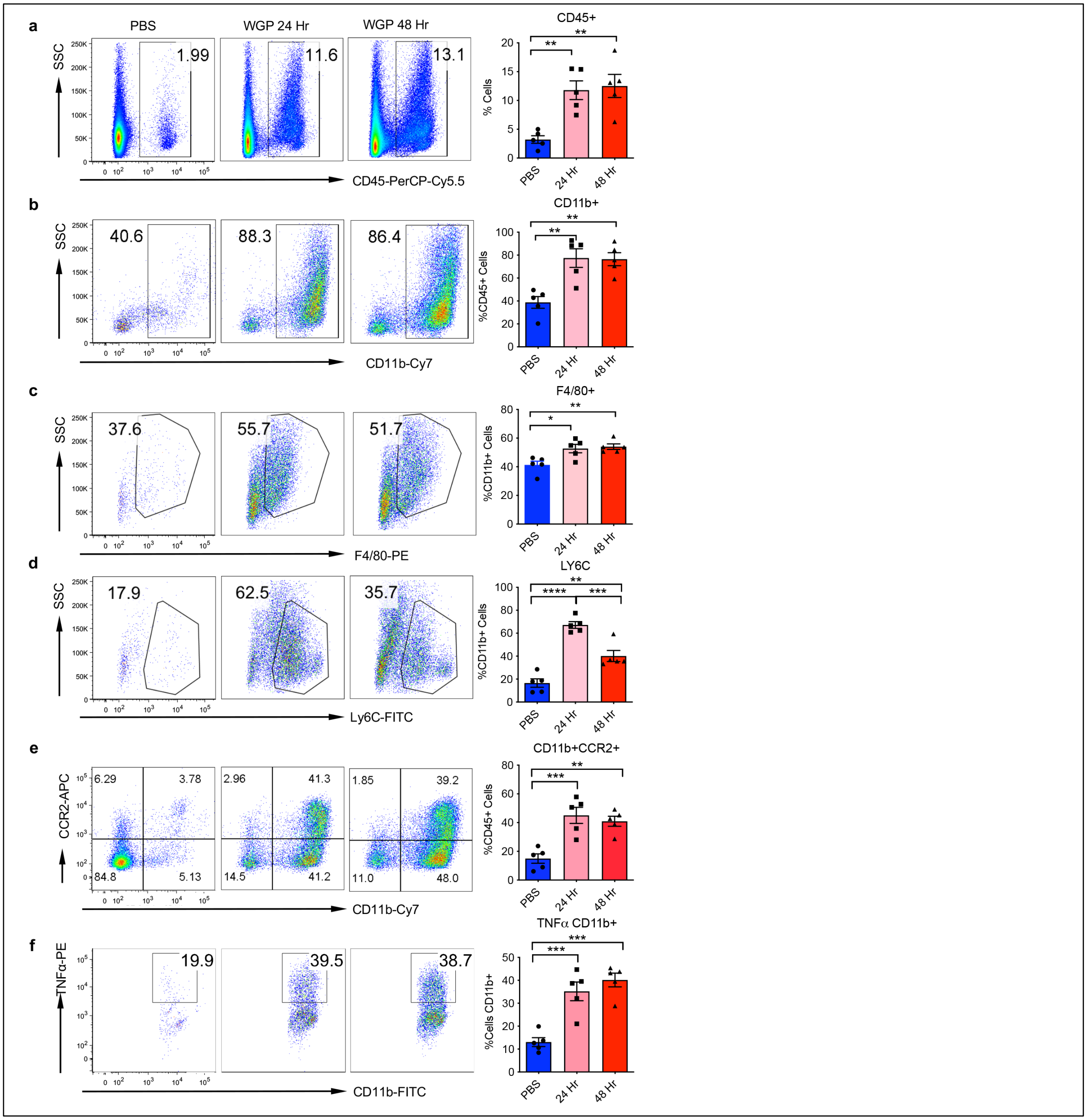

Supplementary Figu. 4: Pancreatic myeloid cells are trained by 24 and 48 hours post-WGP

Mice were treated with WGP 24 hours or 48 hours before euthanization and the percent of **(a)** $CD45^+$, **(b)** percent of $CD45^+$ cells that were $CD11b^+$, **(c)** percent of $CD45^+CD11b^+$ cells that were $F4/80^+$ and **(d)** the percent of $CD45^+CD11b^+$ cells that were $Ly6C^+$ cells were measured and compared to that of a PBS treated mouse. **(e)** Pancreatic cells from PBS and 24 and 48 hour WGP treated mice were gated on the CD45+ population and the percent of these cells that were $CD11b^+CCR2^+$ was assessed. **(f)** Pancreatic cells from PBS and 24 and 48 hour WGP treated mice were restimulated with LPS and the production of TNFα was measured. All analyses were done by flow cytometry and representative and summarized data are shown. Data are represented as mean ± SEM. A one-way ANOVA with multiple comparisons was used. ns= not significant; **p* <.05, ***p* <.01, ****p* <.001, *****p* <.0001

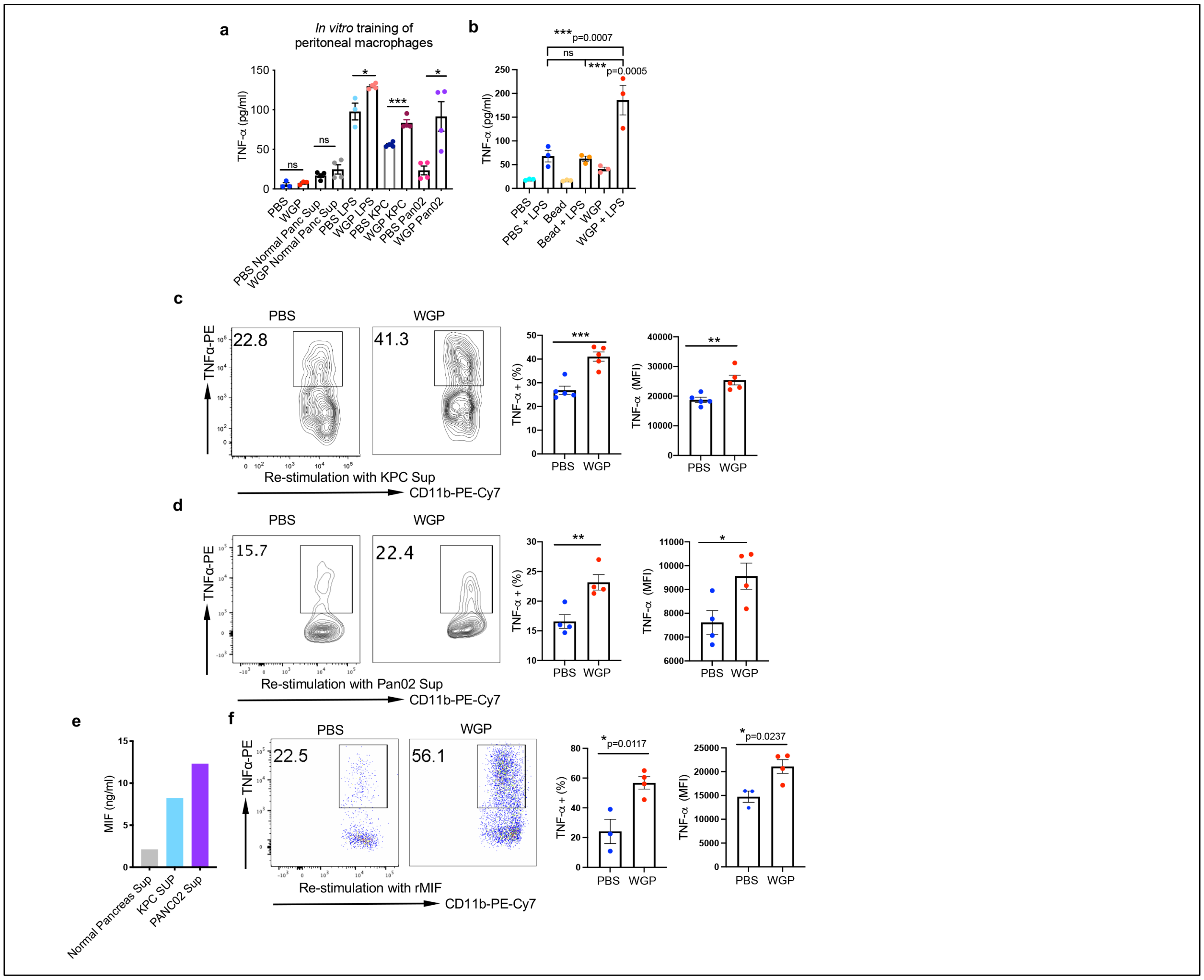

Supplementary Fig. 5: Tumor conditioned media elicits the trained response

(a) Peritoneal macrophages were acquired from WT mice and were stimulated *in vitro* with PBS or WGP for 24 hours. Cells were allowed a resting period of 7 days followed by restimulation with PBS, LPS, the supernatant from cultured and untreated pancreatic cells, or the supernatant from cultured KPC or Pan02 pancreatic cancer cells for 24 hours. The amount of TNFα released over the 24 hours was quantified using an ELISA (PBS n=3, WGP n=3, PBS+ normal pancreatic sup n=3, WGP+ normal pancreatic sup n=4, PBS LPS n=3, WGP LPS n=4, PBS KPC n=4, WGP KPC n=4, PBS Pan02 n=4, WGP Pan02 n=4). *p=0.0175 (PBS LPS vs WGP LPS), ***p=0.0006 (PBS KPC vs WGP KPC), *p=0.0127 (PBS Pan02 vs WGP Pan02). **(b)** Peritoneal macrophages were acquired from WT mice and were stimulated *in vitro* with PBS, WGP, or 3 μm polystyrene beads for 24 hours. Cells were allowed a resting period of 7 days followed by a 24 hour restimulation with PBS or LPS. The amount of TNFα released over the 24 hours was quantified using an ELISA (n=3). ***p=0.0007 (PBS LPS vs WGP LPS), ***p=0.0005 (Bead LPS vs WGP LPS). **(c,d)** WT mice were treated with PBS or WGP and 7 days later pancreatic cells were processed and restimulated with the supernatant from cultured **(c)** KPC (PBS n=5, WGP n=5) **p=0.0065, ***p=0.0006 or **(d)** Pan02 pancreatic cancer cells (PBS n=4, WGP n=4). *p=0.0393, **p=0.0087 **(e)** The amount of rMIF in the supernatant from cultured untreated WT pancreatic cells, KPC cells and Pan02 cells was measured using an ELISA. **(f)** WT mice were treated with PBS or WGP and 7 days later pancreatic cells were processed and restimulated with 10 ng/mL of rMIF (PBS n=3, WGP n=4). *p=0.0117 (percent), *p=0.0237 (MFI). Data are represented as mean ± SEM. A one-way ANOVA with multiple comparisons was used in **b** and an unpaired student's t-test was used in **a**, **c**, **d** and **f**. ns= not significant.

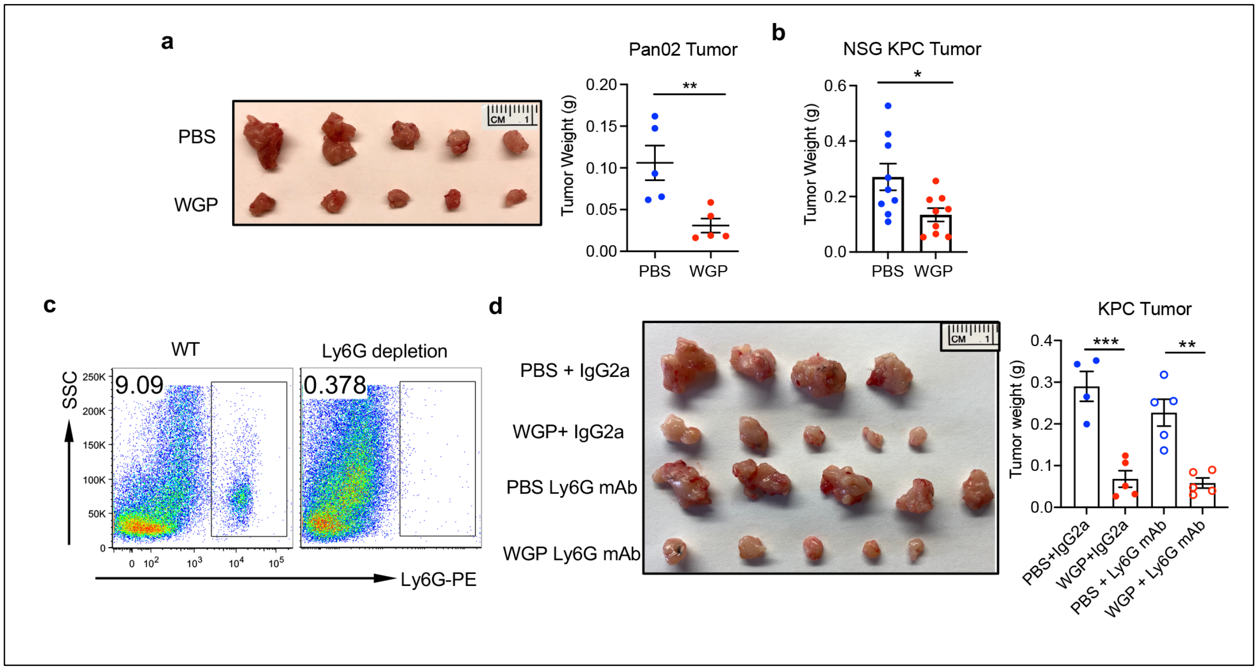

Supplementary Fig. 6: Neither adaptive immune cells nor neutrophils are required for suppression of tumor growth

(a) C57BL/6 mice received a single i.p. injection of WGP or PBS and 7 days mice were implanted orthotopically with Pan02 pancreatic cancer cells. Representative pictures of tumors and quantitative analysis of tumor weight are shown. Tumor weight was recorded at day 21 (PBS n=5, WGP n=5). **p=0.0099 **(b)** NSG mice were treated with either PBS or WGP and 7 days later orthotopic KPC pancreatic tumors were implanted. 21 days following tumor implantation, mice were euthanized and tumor weight was recorded (PBS n=9, WGP n=9). *p=0.0216 **(c,d)** To deplete neutrophils, mice were treated with anti-Ly6G mAb or anti-rat IgG2a isoype control on day -2. On day 0 mice were treated with PBS or WGP and on day 7 mice were implanted with orthotopic KPC pancreatic tumors. Additional doses of the anti-Ly6G mAb or anti-rat IgG2a isoype control were given on days 4, 10 and 16 to maintain the Ly6G depletion. On day 21, mice were euthanized and **(c)** the depletion efficiency was assessed and **(d)** tumor weight was measured (PBS+IgG2a n=4, WGP+IgG2a n=5, PBS + Ly6G mAb n=5, WGP+Ly6G mAb n=5). **p=0.0013, ***p=0.0002. Data are represented as mean ± SEM. An unpaired student's t-test was used for **a** and **b** and a one-way ANOVA with multiple comparisons was used for **d**. ns= not significant.

a

Sample	Initial Cell Count	Knee	Inflection	Filtered Genes	Filtered Cells
AGGREGATE	17,370	10,162	1,013	18,301	11,132
PBS	6,448	10,419	1,199	16,802	4,213
WGP Day 3	5,510	12,513	600	16,340	3,757
WGP Day 7	5,412	8,958	1,418	16,020	3,244

b

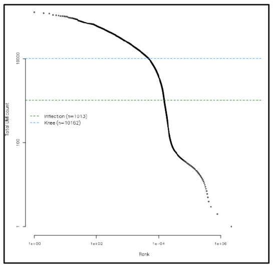

Supplementary Fig. 7: scRNA-Seq
(a) Initial and filtered number of cell barcodes and genes. **(b)** Ranking of call cells by UMI count of PBS, WGP-3day and WGP-7day, aggregated. Each curve has an associated knee and inflection point, which indicates the likely range of true singlet droplet cells.

Supplementary Table 1. Top 20 enriched KEGG pathways for the analyzed comparison (WGP vs PBS)

NAME	SIZE	ES	NES	NOM p-val	FDR q-val
KEGG_HEMATOPOIETIC_CELL_LINEAGE	55	0.568	1.903	0	0.003
KEGG_GLYCOLYSIS_GLUCONEOGENESIS	43	0.578	1.900	0	0.002
KEGG_COMPLEMENT_AND_COAGULATION_CASCADES	43	0.578	1.888	0	0.001
KEGG_PENTOSE_PHOSPHATE_PATHWAY	19	0.640	1.872	0	0.001
KEGG_TOLL_LIKE_RECEPTOR_SIGNALING_PATHWAY	87	0.511	1.785	0	0.004
KEGG_INTESTINAL_IMMUNE_NETWORK_FOR_IGA_PRODUCTION	28	0.560	1.749	0	0.006
KEGG_PRION_DISEASES	29	0.554	1.749	0	0.005
KEGG_LEISHMANIA_INFECTION	52	0.519	1.725	0	0.006
KEGG_ARACHIDONIC_ACID_METABOLISM	35	0.532	1.725	0	0.005
KEGG_PRIMARY_IMMUNODEFICIENCY	33	0.532	1.717	0	0.005
KEGG_ALDOSTERONE_REGULATED_SODIUM_REABSORPTION	33	0.527	1.695	0	0.007
KEGG_NOD_LIKE_RECEPTOR_SIGNALING_PATHWAY	54	0.504	1.693	0	0.006
KEGG_ANTIGEN_PROCESSING_AND_PRESENTATION	39	0.511	1.671	0	0.008
KEGG_VEGF_SIGNALING_PATHWAY	69	0.482	1.664	0	0.008
KEGG_PROTEASOME	43	0.504	1.649	0	0.010
KEGG_CYTOKINE_CYTOKINE_RECEPTOR_INTERACTION	187	0.448	1.649	0	0.009
KEGG_B_CELL_RECEPTOR_SIGNALING_PATHWAY	73	0.475	1.630	0	0.011
KEGG_STARCH_AND_SUCROSE_METABOLISM	23	0.535	1.607	0.003	0.014
KEGG_GALACTOSE_METABOLISM	23	0.524	1.599	0.002	0.014
KEGG_JAK_STAT_SIGNALING_PATHWAY	110	0.441	1.572	0	0.019

Nominal P values were used to determine significance. When the nominal p-value is represented as 0, this means $p<.0001$. The nominal P value was calculated by empirical phenotype-based permutation test.

Supplementary Table 2. Top 20 enriched Gene Ontology (GO) biological processes for the analyzed comparison (WGP vs PBS)

NAME	SIZE	ES	NES	NOM p-val	FDR q-val
GO_PROTEASOMAL_UBIQUITIN_INDEPENDENT_PROTEIN_CATABOLIC_PROCESS	22	0.686	2.043	0	0.005
GO_RESPONSE_TO_PROTOZOAN	16	0.710	2.043	0	0.002
GO_MYD88_DEPENDENT_TOLL_LIKE_RECEPTOR_SIGNALING_PATHWAY	33	0.637	2.028	0	0.001
GO_POSITIVE_REGULATION_OF_INTERLEUKIN_6_PRODUCTION	69	0.594	2.027	0	0.001
GO_FOAM_CELL_DIFFERENTIATION	27	0.645	2.003	0	0.001
GO_RESPONSE_TO_TYPE_I_INTERFERON	69	0.580	1.990	0	0.001
GO_NUCLEUS_LOCALIZATION	21	0.659	1.964	0	0.003
GO_GLYCOLYTIC_PROCESS_THROUGH_FRUCTOSE_6_PHOSPHATE	22	0.657	1.964	0	0.002
GO_NEUROINFLAMMATORY_RESPONSE	52	0.584	1.947	0	0.003
GO_REGULATION_OF_MACROPHAGE_DERIVED_FOAM_CELL_DIFFERENTIATION	21	0.654	1.942	0	0.003
GO_NUCLEAR_MIGRATION	19	0.657	1.940	0	0.003
GO_TUMOR_NECROSIS_FACTOR_BIOSYNTHETIC_PROCESS	20	0.650	1.933	0	0.003
GO_POSITIVE_REGULATION_OF_LIPID_STORAGE	16	0.677	1.932	0	0.003
GO_INTERLEUKIN_6_PRODUCTION	115	0.537	1.928	0	0.003
GO_PODOSOME_ASSEMBLY	18	0.660	1.905	0	0.004
GO_INTERFERON_GAMMA_MEDIATED_SIGNALING_PATHWAY	59	0.558	1.904	0	0.004
GO_ACUTE_PHASE_RESPONSE	28	0.613	1.901	0	0.004
GO_GLIAL_CELL_ACTIVATION	43	0.576	1.892	0	0.005
GO_INTERLEUKIN_1_BETA_PRODUCTION	60	0.555	1.876	0	0.006
GO_EOSINOPHIL_CHEMOTAXIS	15	0.666	1.875	0	0.006

Nominal P values were used to determine significance. When the nominal p-value is represented as 0, this means p<.0001. The nominal P value was calculated by empirical phenotype-based permutation test.

Supplementary Table 3. Materials used and supplier information, including antibodies, chemicals, critical commercial assays, cell lines, oligonucleotides, and software/algorithms

**REAGENT or RESOURCE**	SOURCE	IDENTIFIER
**Antibodies**		
Viability Dye	Invitrogen	Ref #: 65-0865-14
Anti-mouse CD45, 30-F11	BioLegend	Cat # 103129<br>RRID: Ab_893343
Anti-mouse CD11b, M1/70	BioLegend	Cat # 101215<br>RRID:AB_312798
Anti-mouse F4/80, BM8	BioLegend	Cat # 123115<br>RRID: AB_893493
Anti-mouse CD3, 17A2	BioLegend	Cat # 100203<br>RRID:AB_312660
Anti-mouse CD4, GK1.5	BioLegend	Cat # 100411<br>RRID:AB_312696
Anti-mouse CD8, 53-6.7	BioLegend	Cat # 100721<br>RRID:AB_312760
Anti-mouse CD19, 6D5	BioLegend	Cat # 115507<br>RRID:AB_313642
Anti-mouse NK1.1, PK136	BioLegend	Cat # 108709<br>RRID:AB_313396
Anti-mouse Ly6C, HK1.4	BioLegend	Cat # 128005<br>RRID:AB_1186134
Anti-mouse Ly6G, 1A8	BioLegend	Cat # 127607<br>RRID:AB_1186104
Anti-mouse CCR2, 475301	R&D Systems	Cat # FAB5538P<br>RRID:AB_1071841<br>4
Anti-mouse TNFα, MP6-XT22	BioLegend	Cat # 506305<br>RRID:AB_315426
Anti-mouse IFNγ, XMG1.2	BioLegend	Cat # 505807<br>RRID:AB_315401
Anti-Rat IgG1, κ isotype, RTK2071	BioLegend	Cat # 400407<br>RRID:AB_326513
Anti-Rat IgG2b, κ isotype, RTK4530	BioLegend	Cat # 400607<br>RRID:AB_326551
Anti-mouse CD8, 53-6.7	BioLegend	Cat # 100755<br>RRID:AB_2562796
Anti-mouse CD3, 145-2C11	BioLegend	Cat # 100345<br>RRID:AB_2563748
Anti-mouse CD4, RM4-5	BioLegend	Cat # 100561<br>RRID:AB_2562762
Anti-mouse CD11b, M1/70	BioLegend	Cat # 101249<br>RRID:AB_2562797)
Anti-mouse Ly6C, HK1.4	BioLegend	Cat # 128039<br>RRID:AB_2563783
Anti-mouse CD19, 6D5	BioLegend	Cat # 115547<br>RRID:AB_2562806

Anti-mouse IFNγ, XMG1.2	BioLegend	Cat # 505843<br>RRID:AB_2562847
Anti-mouse IL-12/IL-35 p53, 27537	R+D Systems	Cat # MAB 1570<br>RRID:AB_2295829
Anti-mouse IL-17, TC11-18H10.1	BioLegend	Cat # 506935<br>RRID:AB_2562850
Anti-mouse CCR2, 475301R	R+D Systems	Cat # MAB55381R
Anti-mouse Granzyme B, 12F9B65	BioLegend	Cat # 662801<br>RRID:AB_2564373
Anti-mouse TCRγδ, GL3	BioLegend	Cat # 118101<br>RRID:AB_313826
Anti-mouse Tbet, 4B10	BioLegend	Cat # 644825<br>RRID:AB_2563788
Anti-mouse CD107a, 1D4B	BioLegend	Cat # 328635<br>RRID:AB_2563708
Anti-mouse TNFα (141Pr), MP6-XT22	Fluidigm	Product # 3141012B
Anti-mouse CD11c (142 Nd), N418	Fluidigm	Product # 3142003B
Anti-mouse IL-2 (144Nd), JES6-5H4	Fluidigm	Product # 3144002B
Anti-mouse CD69 (145 Nd), H.2F3	Fluidigm	Product #3145005B
Anti-mouse F4/80 (146 Nd), BM8	Fluidigm	Product #3146008B
Anti-mouse CD44 (150Nd), IM7	Fluidigm	Product #3150018B
Anti-mouse Ly-6G (151 Eu), IA8	Fluidigm	Product #3151010B
Anti-mouse CD274 (PD-L1) (153 Eu), 10F.9G2	Fluidigm	Product #3153016B
Anti-mouse FoxP3 (158 Gd), FJK-16S	Fluidigm	Product #3165024A
Anti-mouse CD279 (PD-1) (159 Gd), 29F.1A12	Fluidigm	Product #3159024B
Anti-mouse CD62L (L-Selectin) (160 Gd), MEL-14	Fluidigm	Product #3160008B
Anti-mouse iNOS (161Dy), CXNFT	Fluidigm	Product #3161011B
Anti-mouse CX3CR1 (164DY), SA011F11	Fluidigm	Product #3164023B
Anti-mouse IL-6 (167Er), MP5-20F3	Fluidigm	Product #3167003B
Anti-mouse CD206 (169Tm), C089C3	Fluidigm	Product #3169021B
Anti-mouse C161 (NK1.1)(170Er), PK136	Fluidigm	Product #3170002B
Anti-mouse CD80 (171Yb), 16-10A1	Fluidigm	Product #3171008B
Anti-mouse CD86 (172Yb), GL1	Fluidigm	Product #3172016B
Anti-mouse CD223 (LAG-3) (174Yb), C9B7W	Fluidigm	Product #3174019B
Anti-mouse I-A/I-E (209Bi), M5/114.15	Fluidigm	Product #3209006B
Anti-mouse CD45 (89Y), 30-F11	Fluidigm	Product #3089005B
*InVivo*MAb anti-mouse Anti Ly6G, 1A8	Bio X Cell	BE0075-1<br>RRID:AB_1107721
Rat IgG2a Isotype Control, 2A3	Bio X Cell	BE0089<br>RRID:AB_1107769
*InVivo*MAb anti-mouse PD-L1(B7-H1(10F.9G2)	Bio X Cell	BE0101<br>RRID:AB_1094907<br>3
Rat IgG2b Isotype Control (LTF-2)	Bio X cell	BE0090<br>RRID:AB_1107780
Anti Mouse anti NK1.1 mAb PK136	Made in the Lab of Dr. Jun Yan, University of Louisville	

Anti Mouse anti CD4 mAb GK1.5	Made in the Lab of Dr. Jun Yan, University of Louisville	
Anti Mouse anti CD8 mAb 53-6.72	Made in the Lab of Dr. Jun Yan, University of Louisville	
**Chemicals, Peptides, and Recombinant Proteins**		
Tris(2-carboxyethyl)phosphine hydrochloride (TCEP) solution, pH 7.0 (10x1mL, .5M)	MilliporeSigma	626547
HRP-protector peroxidase stabilizer	Boca Scientific	222050
Antibody Stabilizer PBS	Boca Scientific	131050
Sodium Azide, BioUltra, >99.5%(T)	MilliporeSigma	71289
5-([4,6-Dichlorotriazin-2-yl]amino)fluorescein hydrochloride<br>(DTAF)	Sigma-Aldrich	D0531
ACK	Thermo Fisher Scientific	A1049201
Brefeldin A	Biolegend	420601
Collagenase I	Sigma	C9891
DNase	Sigma	D5025
Endotoxin-free DPBS	Sigma-Aldrich	TMS-012-A
Hyaluronidase	Sigma	H2126
Matrigel® Basement Membrane Matrix, LDEV-free, 5 mL	Corning	356234
Ionomycin Calcium Salt	Sigma-Aldrich	I0634
PMA	Sigma-Aldrich	79346
Fixation Buffer	BioLegend	420801
Permeabilization buffer	BioLegend	421002
Fluoresbrite® YG Microspheres, Calibration Grade 3.00µm	Polysciences	18861-1
3.00µm polystyrene Miroparticle	Sigma-Aldrich	79166
MACS Running Buffer	Miltenyi Biotec	130-091-221
HEPES	Corning	25-060-CI
TRIzol	Invitrogen	15596018
Poly-L-Lysine	Advanced BioMatrix	5048
XenoLight D-Luciferin – K+ Salt Bioluminescent Substrate	Perkin Elmer	12799
CD11b MicroBeads, Human and mouse	Miltenyi Biotec	130-049-601
N-Acetyl-l-cysteine (NAC)	Sigma-Aldrich	A7250-5G
Lipopolysaccharides from E.coli0111:B4	Sigma	L4391-1MG
.25% Trypsin 2.21 mM EDTA, 1x [-] sodium bicarbonate	Corning	25-053-Cl
FBS	Atlanta Biologicals	Cat #: S11150
Bovine Serum Albumin Cohn Fraction V Powder	Equitech0Bio, Inc.	Cat # BAC62-1000<br>Lot #171130-0262
Buprenorphine, hydrochloride, Injection	Par Pharmaceutical	10153802
p-SCN-Bn-Deferoxamine	Macrocyclics	B-705
sodium carbonate	VWR	BJ34277-1L

Oxalic acid, anhydrous	Sigma	756888-50G
UltraPure™ 1 M Tris-HCI Buffer, pH 7.5	Invitrogen	15567027
Zr-89 oxalate	Washington University at St. Louis	Radionuclide Processing Laboratory
Dulbecco's Phosphate Buffered Saline	Sigma	D8537
Chloroform	Sigma	C2432
2-propanol	Fisher Scientific	A41701
UltaPure™ Glycogen	Thermo Fisher Scientific	10814010
iScript™ cDNA Synthesis Kit	BioRad	1708891
iQ™ SYBR® Green Supermix	BioRad	1708880
RPMI-1640 medium	Sigma	R8758-1L
DMEM- high glucose	Sigma	D5796-500ML
**Critical Commercial Assays**		
MaxPar MCP9 Antibody Labeling Kit, 106Cd	Fluidigm	Cat # 201106A
MaxPar MCP9 Antibody Labeling Kit, 111Cd	Fluidigm	Cat # 201111A
MaxPar MCP9 Antibody Labeling Kit, 112Cd	Fluidigm	Cat # 201112A
MaxPar MCP9 Antibody Labeling Kit, 113Cd	Fluidigm	Cat # 201113A
MaxPar MCP9 Antibody Labeling Kit, 114Cd	Fluidigm	Cat # 201114A
MaxPar MCP9 Antibody Labeling Kit, 116Cd	Fluidigm	Cat # 201116A
MaxPar X8 Antibody Labeling Kit, 146Nd	Fluidigm	Cat # 201146A
MaxPar X8 Antibody Labeling Kit, 149 Sm	Fluidigm	Cat # 201149A
MaxPar X8 Antibody Labeling Kit, 154 Sm	Fluidigm	Cat # 201154A
MaxPar X8 Antibody Labeling Kit, 156 Gd	Fluidigm	Cat # 201156A
MaxPar X8 Antibody Labeling Kit, 162 Dy	Fluidigm	Cat # 201162A
MaxPar X8 Antibody Labeling Kit, 165 Ho	Fluidigm	Cat # 201165A
MaxPar X8 Antibody Labeling Kit, 166 Er	Fluidigm	Cat # 201166A
MaxPar X8 Antibody Labeling Kit, 168Er	Fluidigm	Cat # 201168A
MaxPar X8 Antibody Labeling Kit, 173Yb	Fluidigm	Cat # 201173A
ELISA MAX™ standard set Mouse TNF-α	BioLegend	430901
ELISA MAX™ standard set Mouse IL-6	BioLegend	431301
Mouse MIF DuoSet ELISA	R&D Systems	DY1978
Invitrogen pHrodo™ Green S. *aureus* BioParticles™ Phagocytosis Kit for Flow cytometry	Thermo Fisher Scientific	P535367
Amylase Activity Assay Kit	Millipore Sigma	MAK009
QIAGEN RNAeasy Kit	QIAGEN	74104
Promega Luciferase Assay Systems Luciferase Assay w/ Reporter Lysis buffer	Promega	E4030
Chromium Next GEM Single Cell 3′ GEM, Library & Gel Bead Kit v3.1, 4 rxns	10X Genomics	PN-1000128
MiSeq Reagent Micro Kit V2 300 cycles	Illumina	MS-102-2002
NextSeq 500 High Output Kit v2.5 (150 cycles)	Illumina	20024907
**Experimental Models: Cell Lines**		
KPC Cell Line	Ximbio	153474
Pan02 Cell Line	Gift from Dr. Yong Lu at Wake Forest University	

KPC/FC1242 Luc/GFP clone 3	Gift from Dr. Michael Dwinell, PhD at the Medical College of Wisconsin	
Experimental Models: Organisms/Strains		
Mouse: C57BL/6J	The Jackson Laboratory	000664
Mouse: B6.129S4-*Ccr2*^{tm1Ifc}/J (CCR2$^{-/-}$)	The Jackson Laboratory	004999
Mouse: Dectin-1$^{-/-}$	Bred at the University of Louisville	(Saijo et al, 2007)
Mouse: NOD.Cg-*Prkdc*^{scid} *Il2rg*^{tm1Wjl}/SzJ (NSG)	The Jackson Laboratory	005557
Mouse: B6(Cg)-Tyr c-2J (B6 Albino mice)	Gift from Dr. Warawa and bred at the University of Louisville	
**Oligonucleotides**		
Primers for IL-6<br>Forward: AAGACAAAGCCAGAGTCCTTCA<br>Reverse: AGAGCATTGGAAATTGGGGT		
Primers for IL-10<br>Forward: AGTGGAGCAGGTGAAGAGTG<br>Reverse: TTCGGAGAGAGGTACAAACG		
Primers for iNOS<br>Forward: GGTGAAGGGACTGAGCTGTTA<br>Reverse: CAACGTTCTCCGTTCTCTTGC		
Primers for TNF-α<br>Forward: TGTAGCCCACGTCGTAGCAAA<br>Reverse: GCTGGCACCACTAGTTGGTTGT		
**Software and Algorithms**		
CyTOF Workflow	Nowicka M., et al., 2019	https://f1000research.com/articles/6-748/v4
Prism 7	GraphPad	https://www.graphpad.com/scientific-software/prism/<br>RRID: SCR_002798
FlowJo v 10.7.1	TreeStar	https://www.flowjo.com/<br>RRID: SCR_008520
FastQC (v0.11.7)	Andrews, 2015	http://www.bioinformatics.babraham.ac.uk/projects/fastqc/
STAR (v2.6)	Dobin et al., 2013	https://github.com/alexdobin/STAR/
HTSeq (v0.10.0)	Anders et al., 2015	https://pypi.org/project/HTSeq/

DESeq2 (v1.24.0)	Love et al., 2014	https://bioconductor.org/packages/release/bioc/html/DESeq2.html
GSEA	Subramanian et al., 2005	https://www.gsea-msigdb.org/gsea/index.jsp
CellRanger (v3.1.0)	Zheng et al., 2017	https://support.10xgenomics.com/
Seurat (v3.1.1)	Stuart et al., 2019	https://cloud.r-project.org/web/packages/Seurat/index.html
UMAP	Becht et al., 2019	
scater (v1.12.2)	McCarthy et al., 2017	https://bioconductor.org/packages/release/bioc/html/scater.html
DropletUtils (v1.4.3)	Lun et al., 2019	https://bioconductor.org/packages/release/bioc/html/DropletUtils.html
sctransfrom (v0.2.0)	Hafemeister and Satija, 2019	https://github.com/ChristophH/sctransform
MAST (v1.10.0)	Finak et al., 2015	https://www.bioconductor.org/packages/release/bioc/html/MAST.html
**Other**		
Amicon Ultra-.5 centrifugal Filter Unit, .5mL V-bottom, 8-pack 3kDa	Millipore Sigma	UFC500308 (3 kDa)
Amicon Ulrta-.5 centrifugal Filter Unit, .5mL V-bottom, 8-pack 50 kDa	Millipore Sigma	UFC505008
Amicon Ultra-.5 centrifugal Filter Unit, .5mL V-bottom, 8-pack 100 kDa	Millipore Sigma	UFC510008
Eppendorf protein LoBind Tubes, 1.5 mL	Eppendorf	0224431081
polyglycolic acid braided absorbable 5/0 suture	CP Medical	421A
silk braided nonabsorbable 5/0 suture	CP Medical	682S
40µm Nylon, Sterile, DNase/RNase Free Cell Strainers	VWR	10199-655

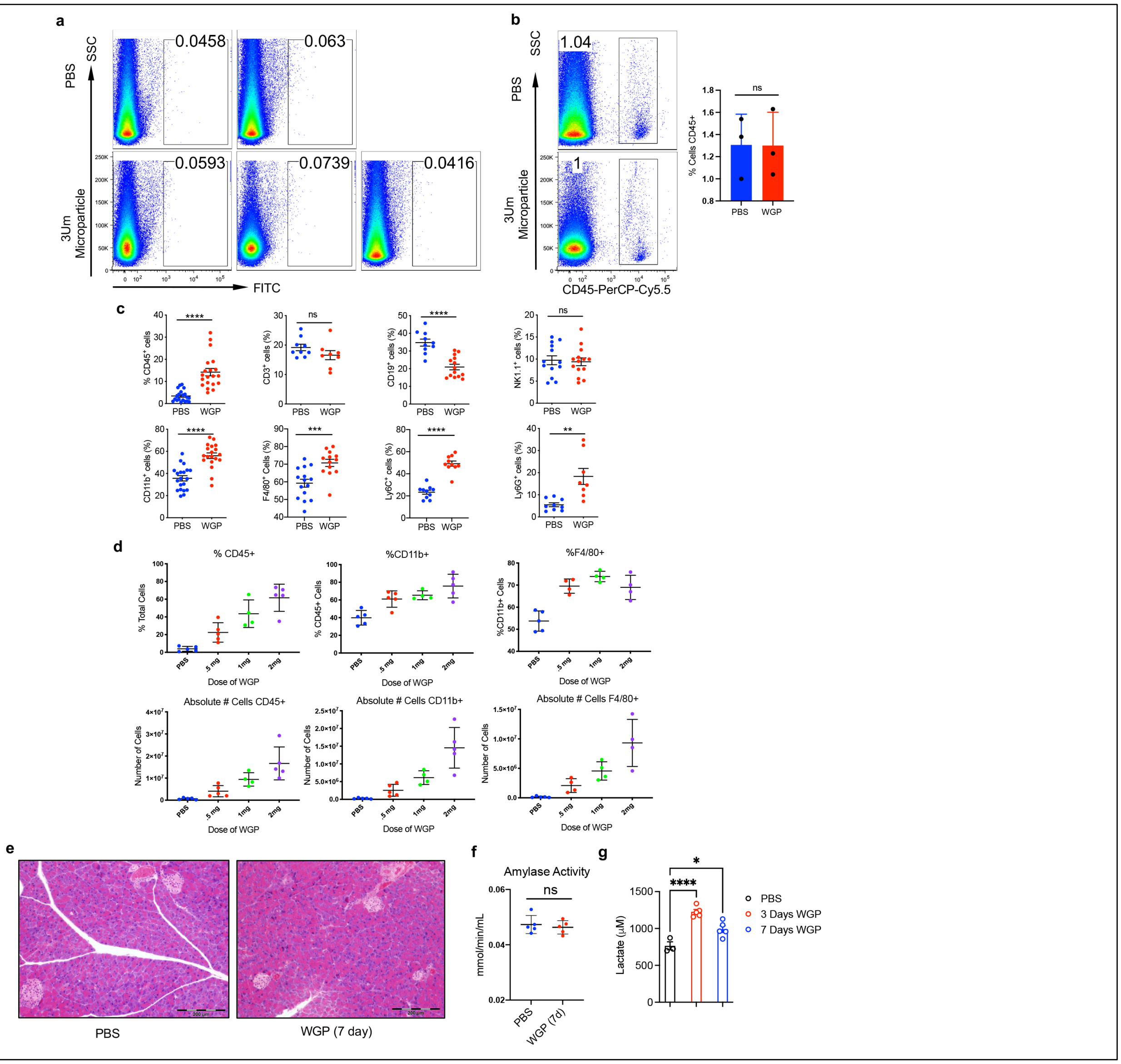

Supplementary Fig. 1: The trafficking of WGP to the pancreas and the associated immune influx are WGP specific and do not cause pancreatic destruction

(a) Fluorescent 3 μm polystyrene beads or PBS was injected IP and 24 hours later the pancreas was assessed for the presence of a fluorescent signal, indicating trafficking. **(b)** 3 μm polystyrene beads or PBS were injected IP and 7 days later the percent of CD45+ cells in the pancreas were assessed. p=0.9789. **(c)** The absolute number of $CD45^+$ immune cells (n=20), $NK1.1^+$ NK cells (n=13), $CD19^+$ B-cells (n=10), $CD3^+$ T-cells (n=8), $CD11b^+$ myeloid cells (n=20), $CD11b^+F4/80^+$ macrophages (n=14), $CD11b^+Ly6C^+$ monocytes (n=10), and $CD11b^+Ly6G^+$ neutrophils (n=8) in PBS and 7-day WGP trained mice. **p=0.0024, ***p=0.0009, ****p<0.0001. **(d)** The percent and absolute number of $CD45^+$, $CD45^+CD11b^+$ and $CD45^+F4/80^+$ cells in PBS mice (n=5) and mice treated with .5 (n=5), 1 (n=4) and 2 (n=5) mg of WGP. **(e)** A representative section from H+E imaging of the pancreas from PBS and 7-day WGP trained mice. **(f)** The levels of amylase in the serum of PBS (n=5) and 7-day WGP (n=5) trained mice. P=0.5928. **(g)** The lactate levels in $CD11b^+$ cell cultures from PBS (n=3), 3-day WGP (n=5), and 7-day (n=5) WGP trained mice. $CD11b^+$ cells were microbeads separated and then restimulated with LPS for 24 h. *p=0.0104, ***p<0.0001. Data are represented as mean ± SEM. An unpaired student's t-test was used for statistical analysis in **b**, **c**, **f**, and a one-way ANOVA with multiple comparisons was used for **g**. ns= not significant.
