# Supplementary material for: The induction of peripheral trained immunity in the pancreas incites anti-tumor activity to control pancreatic cancer progression
Source: Nat Commun. 2022 Feb 9;13:759. doi: 10.1038/s41467-022-28407-4 (PMC8828725; doi:10.1038/s41467-022-28407-4)
Supplement: Supplementary file 3 — Reporting summary [file 41467_2022_28407_MOESM3_ESM.pdf]

nature portfolio

Corresponding author(s): Jun Yan, MD PhD

Last updated by author(s): Dec 20, 2021

Reporting Summary

Nature Portfolio wishes to improve the reproducibility of the work that we publish. This form provides structure for consistency and transparency in reporting. For further information on Nature Portfolio policies, see our Editorial Policies and the Editorial Policy Checklist.

Statistics

For all statistical analyses, confirm that the following items are present in the figure legend, table legend, main text, or Methods section.

n/a	Confirmed	
☐	☒	The exact sample size (n) for each experimental group/condition, given as a discrete number and unit of measurement
☒	☐	A statement on whether measurements were taken from distinct samples or whether the same sample was measured repeatedly
☐	☒	The statistical test(s) used AND whether they are one- or two-sided<br>*Only common tests should be described solely by name; describe more complex techniques in the Methods section.*
☒	☐	A description of all covariates tested
☐	☒	A description of any assumptions or corrections, such as tests of normality and adjustment for multiple comparisons
☐	☒	A full description of the statistical parameters including central tendency (e.g. means) or other basic estimates (e.g. regression coefficient) AND variation (e.g. standard deviation) or associated estimates of uncertainty (e.g. confidence intervals)
☐	☒	For null hypothesis testing, the test statistic (e.g. F, t, r) with confidence intervals, effect sizes, degrees of freedom and P value noted<br>*Give P values as exact values whenever suitable.*
☒	☐	For Bayesian analysis, information on the choice of priors and Markov chain Monte Carlo settings
☒	☐	For hierarchical and complex designs, identification of the appropriate level for tests and full reporting of outcomes
☒	☐	Estimates of effect sizes (e.g. Cohen's d, Pearson's r), indicating how they were calculated

Our web collection on statistics for biologists contains articles on many of the points above.

Software and code

Policy information about availability of computer code

Data collection	FACSDiva version 6 was used to acquire all flow data
Data analysis	Flowjo v10.7.1 to analyze all flow data; Prism 7 for statistical analysis. For RNAseq and sc-RNAseq, publicly available softwares were used: FastQC (v0.11.7), STAR (v2.6), HTSeq (v0.10.0), DESeq2 (v1.24.0), CellRanger (v3.1.0), Seurat (v3.1.0), scater (v1.12.2), DropletUtils (v1.4.3), sctransfrom (v0.2.0), and MAST (v1.10.0).

For manuscripts utilizing custom algorithms or software that are central to the research but not yet described in published literature, software must be made available to editors and reviewers. We strongly encourage code deposition in a community repository (e.g. GitHub). See the Nature Portfolio guidelines for submitting code & software for further information.

Data

Policy information about availability of data

All manuscripts must include a data availability statement. This statement should provide the following information, where applicable:

- Accession codes, unique identifiers, or web links for publicly available datasets
- A description of any restrictions on data availability
- For clinical datasets or third party data, please ensure that the statement adheres to our policy

The data generated in this study are available in the data source files. RNASeq data is available under GEO accession GSE187464, and scRNASeq data is available under GEO accession GSE187464 (https://www.ncbi.nlm.nih.gov/geo/)

Field-specific reporting

Please select the one below that is the best fit for your research. If you are not sure, read the appropriate sections before making your selection.

☒ Life sciences ☐ Behavioural & social sciences ☐ Ecological, evolutionary & environmental sciences

For a reference copy of the document with all sections, see nature.com/documents/nr-reporting-summary-flat.pdf

Life sciences study design

All studies must disclose on these points even when the disclosure is negative.

Sample size	For in vitro and in cell culture experiments, 3-5 mice in each group were used. This was based on our pilot experiments. No sample size calculation was performed. Sample sizes were estimated based on standards of the field. For tumor studies, 6-15 mice in each group were used which enable us to detect effect size of 0.9 with power at 80% and a significant level at 5%.
Data exclusions	No data exclusions
Replication	All results were repeated at least three independent times unless otherwise specified. Similar data were obtained in independent experiments.
Randomization	Mice were randomized based on the age and sex
Blinding	Blinding is not possible since the treatment effect was so obvious

Reporting for specific materials, systems and methods

We require information from authors about some types of materials, experimental systems and methods used in many studies. Here, indicate whether each material, system or method listed is relevant to your study. If you are not sure if a list item applies to your research, read the appropriate section before selecting a response.

Materials & experimental systems

n/a	Involved in the study	
☐	☒	Antibodies
☐	☒	Eukaryotic cell lines
☒	☐	Palaeontology and archaeology
☐	☒	Animals and other organisms
☒	☐	Human research participants
☒	☐	Clinical data
☒	☐	Dual use research of concern

Methods

n/a	Involved in the study	
☒	☐	ChIP-seq
☐	☒	Flow cytometry
☒	☐	MRI-based neuroimaging

Antibodies

Antibodies used

Antibodies
Viability Dye Invitrogen Ref #: 65-0865-14
Anti-mouse CD45, 30-F11 BioLegend Cat # 103129 RRID: Ab_893343
Anti-mouse CD11b, M1/70 BioLegend Cat # 101215 RRID:AB_312798
Anti-mouse F4/80, BM8 BioLegend Cat # 123115 RRID: AB_893493
Anti-mouse CD3, 17A2 BioLegend Cat # 100203 RRID:AB_312660
Anti-mouse CD4, GK1.5 BioLegend Cat # 100411 RRID:AB_312696
Anti-mouse CD8, 53-6.7 BioLegend Cat # 100721 RRID:AB_312760
Anti-mouse CD19, 6D5 BioLegend Cat # 115507 RRID:AB_313642
Anti-mouse NK1.1, PK136 BioLegend Cat # 108709 RRID:AB_313396
Anti-mouse Ly6C, HK1.4 BioLegend Cat # 128005 RRID:AB_1186134
Anti-mouse Ly6G, 1A8 BioLegend Cat # 127607 RRID:AB_1186104
Anti-mouse CCR2, 475301 R&D Systems Cat # FAB5538P RRID:AB_10718414
Anti-mouse TNF, MP6-XT22 BioLegend Cat # 506305 RRID:AB_315426
Anti-mouse IFN, XMG1.2 BioLegend Cat # 505807 RRID:AB_315401
Anti-Rat IgG1, isotype, RTK2071 BioLegend Cat # 400407 RRID:AB_326513
Anti-Rat IgG2b, isotype, RTK4530 BioLegend Cat # 400607 RRID:AB_326551
Anti-mouse CD8, 53-6.7 BioLegend Cat # 100755 RRID:AB_2562796
Anti-mouse CD3, 145-2C11 BioLegend Cat # 100345 RRID:AB_2563748
Anti-mouse CD4, RM4-5 BioLegend Cat # 100561 RRID:AB_2562762

Anti-mouse CD11b, M1/70 BioLegend Cat # 101249 RRID:AB_2562797)
Anti-mouse Ly6C, HK1.4 BioLegend Cat # 128039 RRID:AB_2563783
Anti-mouse CD19, 6D5 BioLegend Cat # 115547 RRID:AB_2562806
Anti-mouse IFN, XMG1.2 BioLegend Cat # 505843 RRID:AB_2562847
Anti-mouse IL-12/IL-35 p53, 27537 R+D Systems Cat # MAB 1570 RRID:AB_2295829
Anti-mouse IL-17, TC11-18H10.1 BioLegend Cat # 506935 RRID:AB_2562850
Anti-mouse CCR2, 475301R R+D Systems Cat # MAB55381R
Anti-mouse Granzyme B, 12F9B65 BioLegend Cat # 662801 RRID:AB_2564373
Anti-mouse TCR, GL3 BioLegend Cat # 118101 RRID:AB_313826
Anti-mouse Tbet, 4B10 BioLegend Cat # 644825 RRID:AB_2563788
Anti-mouse CD107a, 1D4B BioLegend Cat # 328635 RRID:AB_2563708
Anti-mouse TNF (141Pr), MP6-XT22 Fluidigm Product # 3141012B
Anti-mouse CD11c (142 Nd), N418 Fluidigm Product # 3142003B
Anti-mouse IL-2 (144Nd), JES6-5H4 Fluidigm Product # 3144002B
Anti-mouse CD69 (145 Nd), H.2F3 Fluidigm Product #3145005B
Anti-mouse F4/80 (146 Nd), BM8 Fluidigm Product #3146008B
Anti-mouse CD44 (150Nd), IM7 Fluidigm Product #3150018B
Anti-mouse Ly-6G (151 Eu), IA8 Fluidigm Product #3151010B
Anti-mouse CD274 (PD-L1) (153 Eu), 10F.9G2 Fluidigm Product #3153016B
Anti-mouse FoxP3 (158 Gd), FJK-16S Fluidigm Product #3165024A
Anti-mouse CD279 (PD-1) (159 Gd), 29F.1A12 Fluidigm Product #3159024B
Anti-mouse CD62L (L-Selectin) (160 Gd), MEL-14 Fluidigm Product #3160008B
Anti-mouse iNOS (161Dy), CXNFT Fluidigm Product #3161011B
Anti-mouse CX3CR1 (164DY), SA011F11 Fluidigm Product #3164023B
Anti-mouse IL-6 (167Er), MP5-20F3 Fluidigm Product #3167003B
Anti-mouse CD206 (169Tm), C089C3 Fluidigm Product #3169021B
Anti-mouse C161 (NK1.1)(170Er), PK136 Fluidigm Product #3170002B
Anti-mouse CD80 (171Yb), 16-10A1 Fluidigm Product #3171008B
Anti-mouse CD86 (172Yb), GL1 Fluidigm Product #3172016B
Anti-mouse CD223 (LAG-3) (174Yb), C9B7W Fluidigm Product #3174019B
Anti-mouse I-A/I-E (209Bi), M5/114.15 Fluidigm Product #3209006B
Anti-mouse CD45 (89Y), 30-F11 Fluidigm Product #3089005B
(The amount of above antibodies used for staining was based on the commercial vendors' recommendation)
InVivoMAb anti-mouse Anti Ly6G, 1A8 Bio X Cell BE0075-1 RRID:AB_1107721
Rat IgG2a Isotype Control, 2A3 Bio X Cell BE0089 RRID:AB_1107769
InVivoMAb anti-mouse PD-L1(B7-H1(10F.9G2) Bio X Cell BE0101 RRID:AB_10949073
Rat IgG2b Isotype Control (LTF-2) Bio X cell BE0090 RRID:AB_1107780
Anti-tri-methyl-histone H3 (lys4) rabbit mAb (C42D8), Cell Signaling, Cat#9751, 1:1000
Anti-acetyl-histone H3 (lys27) XP rabbit mAb (D5E4), Cell Signaling, Cat#8173, 1:1000
Anti-tri-methyl-histone H3 (Lys27) rabbit mAb (C36B11), Cell Signaling, Cat#9733, 1:1000
Anti-histone H3 XP rabbit mAb (D1H2), Cell Signaling, Cat#4499, 1:1000
HRP-conjugated secondary antibody (from donkey), GE Healthcare, Code# NA934, 1:3000 dilution
Anti Mouse anti NK1.1 mAb PK136 Made in the Lab of Dr. Jun Yan, University of Louisville
Anti Mouse anti CD4 mAb GK1.5 Made in the Lab of Dr. Jun Yan, University of Louisville
Anti Mouse anti CD8 mAb 53-6.72 Made in the Lab of Dr. Jun Yan, University of Louisville

Validation

All purchased antibodies were used according to the manufacturer's instructions. Antibodies created within the lab were internally validated against an appropriate isotype control.

Eukaryotic cell lines

Policy information about cell lines

Cell line source(s)

KPC cell line was purchased from Ximbio, Pan02 was a gift from Dr. Yong Lu at Wake Forest University, and KPC/FC1242 Luc/GFP clone 3 was a gift from Dr. Michael Dwinell from Medical College of Wisconsin.

Authentication

Pathogen detection for KPC cell line was performed by VRL laboratory and non of other two cell lines were authenticated.

Mycoplasma contamination

Cells were confirmed mycoplasma negative using ATCC Mycoplasma detection kit

Commonly misidentified lines
(See ICLAC register)

None of the cell lines used in the study is listed as Commonly misidentified lines.

Animals and other organisms

Policy information about studies involving animals; ARRIVE guidelines recommended for reporting animal research

Laboratory animals

C57Bl/6 mice, Dectin-1-/-, CCR2 KO mice, NSG mice, 6-8 weeks old, both sexes. All mice were housed under SPF condition with free food and water supply with 12 h dark/light cycle at room temperature with controlled humidity (around 55%).

Wild animals	This study did not involve wild animals
Field-collected samples	No animals were from field
Ethics oversight	The University of Louisville IACUC committee approved this study

Note that full information on the approval of the study protocol must also be provided in the manuscript.

Flow Cytometry

Plots

Confirm that:

- [x] The axis labels state the marker and fluorochrome used (e.g. CD4-FITC).
- [x] The axis scales are clearly visible. Include numbers along axes only for bottom left plot of group (a 'group' is an analysis of identical markers).
- [x] All plots are contour plots with outliers or pseudocolor plots.
- [x] A numerical value for number of cells or percentage (with statistics) is provided.

Methodology

Sample preparation	Pancreas samples were digested to make a single cell suspension. For regular flow analysis, cells were blocked with Fc blocker and then stained with fluorochrome-labeled mAbs and relevant isotype controls. Viability dye was added to distinguish dead cells. These have been described in depth in the methods of the paper.
Instrument	FACSCanto II cytometer (BD Biosciences)<br>FACS Aria III (Bd Biosciences)
Software	FlowJo V. 7.10.1 made by TreeStar was used for analysis of flow cytometry data.
Cell population abundance	If sorting was used, purify was always >90%. These have been described in depth in the methods of the paper.
Gating strategy	We used viability dye to gate out dead cells and then use CD45 to gate all immune cells. Then different subsets of cells were gated from CD45+ cells. These have been described in depth in the methods of the paper.

- [x] Tick this box to confirm that a figure exemplifying the gating strategy is provided in the Supplementary Information.
